# Psychological symptoms as the initial manifestation of choline kinase β-related muscular dystrophy

**DOI:** 10.1515/jtim-2026-0021

**Published:** 2026-04-04

**Authors:** Yakun Wu, Yanyu Lu, Chang Liu, Yajie Wang, Xujun Chu, Zhaoxia Wang, Yun Yuan, Zhiying Xie

**Affiliations:** Department of Neurology, Peking University First Hospital, Beijing, China; Department of Neurology, Tangshan Gongren Hospital, Tangshan, Hebei Province, China; Beijing Key Laboratory of Neurovascular Disease Discovery, Beijing, China

## To the editor

Megaconial congenital muscular dystrophy (CMD) was first described in 1998, and is characterized by early-onset muscle weakness and the presence of giant, megaconial mitochondria within muscle fibers. Subsequently, Nishino *et al*. identified mutations in choline kinase β (*CHKB*) gene in 15 affected patients, establishing the molecular basis of this CMD subtype.^[1]^ *CHKB* encodes an enzyme essential for the biosynthesis of phosphatidylcholine, a key structural lipid in cellular membranes.^[2]^ Later studies expanded the phenotype spectrum of this multisystem disorder, identifying limb gridle muscular dystrophy (LGMD) as a rarer, milder presentation of *CHKB*-related muscular dystrophy (MD). Core clinical features include progressive muscle weakness, central nervous system involvement, cardiomyopathy, and ichthyosis. Despite phenotypic variation among Chinese patients, juvenile-onset LGMD has not previously been reported. Here, we describe a child whose initial symptoms involved behavioral disturbances preceding mild limb-girdle weakness, along with a comprehensive review of *CHKB*-related MD.

The patient, a 9-year-4-month-old boy, exhibited normal cognitive and motor milestones during early childhood. Behavioral abnormalities emerged at age 5, characterized by temper tantrums, aggression, and reduced social engagement. By age 7, he developed progressive lower limb weakness. Manual muscle testing revealed grade 4/5 strength in shoulder adduction and abduction, elbow extension, and hip flexion. Serum creatine kinase levels were elevated at 940 U/L, and electromyography revealed myopathic changes. Brain magnetic resonance imaging (MRI), electrocardiogram, and echocardiography were normal.

**Figure 1:**
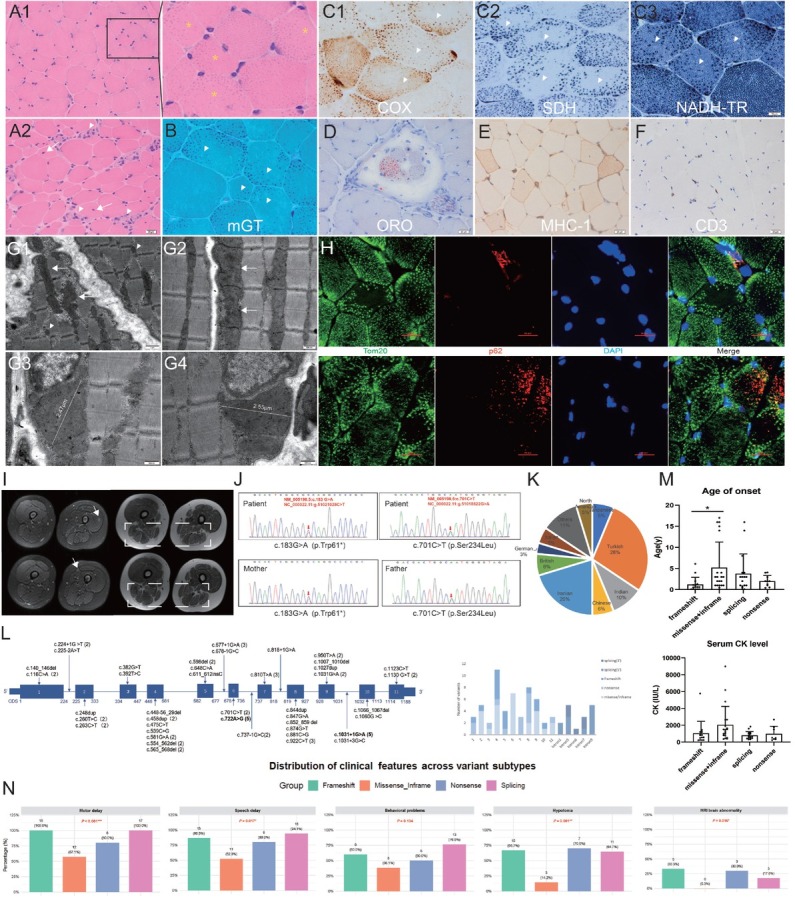
Clinical, imaging, and biopsy findings in the reported patient with CHKB-related muscular dystrophy. (A) HE staining revealed necrosis, regeneration (arrowheads), internal nuclei (arrows), and coarse basophilic granules (yellow stars). (B-C) mGT (B), COX, SDH, and NADH-TR (C1-3) staining demonstrated peripherally distributed mitochondria in some muscle fibers (arrowheads). (D) Oil Red O staining showed lipid droplet accumulation in muscle spindle fibers. (E-F) MHC-I and CD3 immunohistochemistry. Ultrastructural analysis (G) and immunofluorescence staining for mitochondrial marker Tom20 (H) confirmed conically enlarged mitochondria beneath the sarcolemma (arrows for enlarged mitochondria, arrowheads for normal mitochondria). (H) Immunofluorescence staining for p62 was positive in some fibers. (I) MRI T1 showed mild fatty infiltration in the posterior compartment of the thigh (dashed box). IDEAL axial thigh images showed mild muscle edema (arrows) in the quadriceps femoris. (J) Sanger sequencing results of the patient and his parents. (K) Geographic distribution of CHKB-related MD cases. (L) Schematic of the CHKB gene and distribution of variants across exons and introns. (M) The Kruskal-Wallis test revealed a significant difference in age at onset, but not in serum creatine kinase levels, across the variant groups. Post-hoc pairwise comparisons using the Mann-Whitney *U* test showed that age at onset was significantly later in the missense/in-frame group than in the frameshift group (*P* = 0.016). (N) CMD features were compared across variant groups using the Chi-square test for hypotonia and the Fisher-Freeman-Halton exact test for motor delay, speech delay, behavioral problems, and MRI brain abnormalities. ^*^P < 0.05; ^**^P < 0.01; ^***^P < 0.001. HE: hematoxylin and eosin; mGT: modified Gomori trichrome; COX: cytochrome c oxidase; SDH: succinate dehydrogenase; NADH-TR: nicotinamide adenine dinucleotide–tetrazolium reductase; EM: electron microscope; IDEAL: iterative decomposition of water and fat with echo asymmetry and least-squares estimation.

Muscle biopsy revealed atrophic fibers, endomysial proliferation, necrosis, regeneration, and internal nuclei (Figure 1A), consistent with nonspecific dystrophic changes. Coarse basophilic granules with peripheral localization (HE, mGT) (Figure 1A and 1B) were identified as mitochondria using mitochondrial enzyme staining (SDH, NADH, COX) and mitochondrial marker Tom20 (Figure 1C and 1H). Transmission electron microscopy confirmed enlarged, conical mitochondria with disorganized cristae predominantly located beneath the sarcolemma (Figure 1G). Lipid droplet accumulation was evident in muscle spindle fibers (Figure 1D). Immunohistochemistry demonstrated upregulation of major histocompatibility complex (MHC) class I and infiltration of CD3^+^ lymphocytes (Figure 1E and 1F). Immunofluorescent examination showed the presence of fibers with intense positivity for p62, indicating autophagy activation (Figure 1H). Magnetic resonance imaging (MRI) of the thighs revealed moderate fatty infiltration in the adductor magnus and semitendinosus, along with mild edema in the vastus lateralis and medialis (Figure 1I).

Genetic analysis identified compound heterozygous *CHKB* variants—c.701C>T (p.Ser234Leu) and c.183G>A (p.Trp61*)—in the patient. The c.701C>T variant was inherited from the father and c.183G>A from the mother (Figure 1J). The c.183G>A variant introduces a premature stop codon at tryptophan 61, truncating the protein upstream of the choline kinase domain in the N-terminal region and resulting in near-complete loss of function. This variant has not been previously reported. The population allele frequency of c.183G>A in the Genome Aggregation Database was extremely low (0.00001591; 0.00001647 in ExAC), while c.701C>T was absent. Both variants were predicted to be deleterious by multiple in silico tools, fulfilling PP3 criteria. According to the 2015 ACMG–AMP guidelines, both were classified as pathogenic (Supplementary Table S1).

To date, 63 *CHKB*-related MD cases have been reported across 18 countries,^[3, 4, 5, 6, 7, 8, 9, 10]^ with over 60% originating from Asia (Figure 1K). A total of 42 pathogenic *CHKB* variants have been documented (Supplementary Table S2). Most patients (87.3%) carry homozygous variants. Variants are distributed across all 11 exons, though exon 4 exhibits relatively higher variant frequency. Common variants include c.722A>G and c.1031+1G>A, without an apparent mutational hotspot. Splicing variants occur primarily at canonical splice sites, with a higher frequency at the 5′donor site compared to the 3′acceptor site (ratio 13:5) (Figure 1L).

Patients were categorized into four variant-based subgroups: frameshift, missense/in-frame, splicing, and nonsense (Supplementary Table S3). Symptom onset occurred before age 3 in over 75% of cases. Frameshift variants were associated with the earliest age of onset (median 0.6 years, IQR 0.1–1.3), while missense/in-frame variants had the latest age of onset (median 3.0 years, IQR 1.0–9.0) (*P* = 0.016) (Figure 1M). Typical CMD features included motor delay, speech delay, and hypotonia. The frequency of CMD features varied significantly among subgroups, with missense/in-frame variants exhibiting a milder presentation (Figure 1N). However, the overall prevalence of intellectual disability (88.9%) did not differ among subgroups.

Common comorbidities included behavioral abnormalities (55.6%), dermatologic findings (39.7%), seizures (20.1%), and gastrointestinal symptoms (12.7%). Approximately 22% of patients exhibited cardiac involvement; while the missense/in-frame subgroup showed the lowest prevalence (9.5%), this difference was not statistically significant. Additional features included sleep disturbances, hearing loss, and dysmorphic facial characteristics. Acute intercurrent infections, vaccination, or general anesthesia were reported to exacerbate myopathy and/or cardiomyopathy in 11.1% of patients. Serum creatine kinase levels were typically mildly elevated or normal (median 893.5 IU/L, IQR 476.8–1500), with the highest levels observed in the missense/in-frame group (*P* = 0.05) (Figure 1M). Brain MRI abnormalities, including corpus callosum thinning and ventricular enlargement, were noted in 17.4% of patients but absent in the missense/in-frame subgroup (Figure 1N). Muscle MRI generally revealed diffuse fatty infiltration and atrophy of thigh muscles, with relative preservation of the adductor longus and calf muscles.

We report a Chinese patient with a novel clinical phenotype associated with compound heterozygous *CHKB* variants. The abnormal mitochondria and lipid droplet accumulation observed in the muscle fibers of the reported patient reflect the underlying metabolic dysfunction characteristic of *CHKB*-related MD. Our literature review highlights that the *CHKB* genotype has a strong influence on clinical variability.

## Supplementary Material

Supplementary Material Details
