# Supplementary material for: Psychological symptoms as the initial manifestation of choline kinase β-related muscular dystrophy
Source: J Transl Int Med. 2026 Apr 4;14(2):326–9. doi: 10.1515/jtim-2026-0021 (PMC13110456; doi:10.1515/jtim-2026-0021)
Supplement: Supplementary file 1 — Supplementary Material Details [file jtim-2026-0021_sm.pdf]

Supplementary materials

Psychological symptoms as the initial manifestation of choline kinase β-related muscular dystrophy

Yakun Wu[1,2#], Yanyu Lu[1#], Chang Liu[1], Yajie Wang[1,3], Xujun Chu[1], Zhaoxia Wang[1,3], Yun Yuan[1,3], Zhiying Xie[1]

[1]Department of Neurology, Peking University First Hospital, Beijing 100034, China

[2]Department of Neurology, Tangshan Gongren Hospital, Tangshan 063000, Hebei Province, China

[3]Beijing Key Laboratory of Neurovascular Disease Discovery, Beijing 100034, China

Address for Correspondence: Zhiying Xie, Department of Neurology, Peking University First Hospital, No. 8 Xishiku Street, Xicheng District, Beijing 100034, China. E-mail: xiezhiyingxzy@163.com

Supplementary Table S1. Genetic data and pathogenicity evaluation of *CHKB* variants identified in the reported patient with LGMD

Exon	c.DNA position	Protein prediction	Parental derivation	Global AF in all subpopulation			Silico analysis				GERP*	Variants pathogenicity	Evidence of pathogenicity
				gnomAD	TGP	ExAC	REVEL	Mutation Taster	PolyPhen2	SIFT			
Exon 6	c.701 C>T	p.Ser234Leu	Paternal	absent	absent	absent	-	disease causing automatic	-	-	5.01	Pathogenic	PS1, PM2, PM3, PP1, PP3, PP4
Exon 1	c.183 G>A	p.Trp61*	Maternal	0.00001591	absent	0.00001.647	0.570	disease causing	deleterious	tolerated	5.41	Pathogenic	PVS1, PM2, PP1, PP3, PP4

*, The cutoff was set to 2.0 for GERP (smaller scores indicating less conservation). AF, allele frequency; gnomAD, Genome Aggregation Database; TGP, 1000 Genomes Project; ExAC, Exome Aggregation Consortium Browser. *CHKB* variants were described in relation to the *CHKB* gene genomic reference sequence (NG_029213.1), coding DNA reference sequence (NM_005198.5) and protein reference sequence (NP_005189.2). PVS, pathogenic very strong; PS, pathogenic strong; PM, pathogenic moderate; PP, pathogenic supporting.

Supplementary Table S2. The clinical data of patients with *CHKB*-related Muscular Dystrophy

No.	Author	Origin	Gender	Motor delay	Speech delay	Intellectual disability	Behavioral problems	Cardiac abnormalities	Skin abnormalities	Seizure	Bowel and bladder involvement	Joint contractures	Hypotonia	Facial weakness	Other features	CK level (IU/L)	cDNA	Protein	Muscle biopsy	MRI brain	Muscle MRI
1	Nishino I, *et al* 1998	Janpanese	F	+	NA	+	NA	DCM	-	-	NA	NA	+	NA		370	Hom c.810T>A	p.Tyr270*	+	normal	NA
2			M	+	+	+	-	+	-	+	NA	NA	+	NA		465-2676	Hom c.810T>A	p.Tyr270*	NA	mild brain atrophy	NA
3			F	+	+	+	NA	+	-	+	NA	NA	+	+	precocious sexual	502	c.116C>A + c.458dup	p.Ser39* + p.Leu153Phefs*57	NA	normal	NA
4			M	+	+	+	NA	-	-	+	NA	NA	+	+		230	c.116C>A + c.458dup	p.Ser39* + p.Leu153Phefs*57	NA	normal	NA
6	Gutiérrez Ríos P, *et al* 2012	African-American	M	+	+	+	+	-	NA	-	NA	NA	+	+		318-522	Hom c.874G>T	p.Glu292*	+	prominent CSF spaces, and ventricular enlargements	NA
7	Quinlivan R, *et al* 2013	Bristish	M	+	+	+	autism	severe DCM	ichthyosis	NA	feeding difficulty, recurrent abdominal pain	-	-	-	transient loss of leg movement following a routine vaccination/chicken pox	611-1780	Hom c.852_859 del	p.Trp284*	+	normal	NA
8			F	-	+	+	NA	LVD	-	NA	NA	-	NA	-	loss of ambulation after episodes of pancreatitis complicated by ARDS	462	c.722A>G + c.881C>G	p.Asn241Ser + p.Pro294Arg	+	normal	extensive fatty replacement
9			M	+	+	+	NA	-	ichthyosis	NA	NA	NA	NA	-	episode of increased falling after an URTI	1000	Hom c.722A>G	p.Asn241Ser	+	normal	NA
10	Mitsuhashi S, *et al* 2013	French	M	+	+	+	NA	NA	NA	NA	NA	NA	NA	NA		High	Hom c.581G>A	p.Arg194Gln	+	NA	NA
11	Castro-Gago M, *et al* 2014	Spanish	M	-	-	+	+	-	NA	NA	NA	NA	+	-		250	Hom c.810T>A	p.Tyr270*	+	normal	NA
12	CABRERA-SERRANO M, *et al* 2015	Australian	F	+	+	+	NA	-	NA	NA	NA	NA	+	-	deterioration of muscle strength during intercurrent illness	1500	Hom c.648C>A	p.Tyr216*	+	normal	NA
13	Haliloglu G, *et al* 2015	Turkish	M	+	+	severe	autism	DLVSF	ichthyosis	-	NA	-	+	FF		843	Hom c.611_612insC	p.Thr205Asnfs*5	+	thinning of CC, prominent frontal horns of LV	NA
14			M	+	+	NA	-	DCM	-	-	NA	-	+	-		258	Hom c.922C>T	p.Gln308*	+	cerebral atrophy, dilatation of ventricles	NA
15			M	+	+	moderate	hyperactivity	-	ichthyosis	-	NA	-	-	-		normal	Hom c.922C>T	p.Gln308*	+	normal	NA
16			F	+	+	NA	-	PDA	hirsutism	-	NA	-	+	FF	microcephaly	368	Hom c.847G>A	p.Glu283Lys	+	normal	NA
17			M	+	+	mild	-	-	-	-	NA	-	-	-		1122	Hom c.1130 G>T	p.Arg377Leu	+	NA	NA
18			F	+	+	severe	autism	-	ichthyosis	+	NA	-	-	-		2669	Hom c.554_562del	p.Pro185_Trp187del	+	NA	NA
19			F	+	+	severe	autism	ASD	ichthyosis	+	NA	-	-	-		1103	Hom c.667+1G>A	p.Gly223Asp	+	normal	NA
20			F	+	+	moderate	-	-	ichthyosis	-	NA	-	+	FF		467	Hom c.667+1G>A	p.Gly223Asp	+	normal	NA
21			F	+	+	mild	Rett, autistic	MVP	-	-	NA	-	-	FF		497	Hom c.667+1G>A	p.Gly223Asp	+	delay in myelination	NA
22			M	+	+	mild	autistic	DLVSF	atopic dermatitis	-	NA	-	-	FF		428	Hom c.1031+1G>A	Splice site affected	+	normal	NA
23			F	+	+	mild	-	NA	ichthyosis	-	NA	-	+	-	sleep disturbance	1606	Hom c.1031+1G>A	Splice site affected	+	normal	NA
24			F	+	+	severe	-	-	ichthyosis	+	NA	-	+	-		normal	Hom c.922C>T	p.Gln308*	+	NA	NA
25			M	+	+	mild	-	NA	ichthyosis	-	NA	-	+	-		600	Hom c.1031+1G>A	Splice site affected	+	NA	NA
26			F	+	+	mild	-	-	ichthyosis	-	NA	-	-	FF		500	c.475C>T + c.1031+1G>A	p.Arg159* + Splice site affected	+	superior vermian prominent folia	NA
27			M	+	+	mild	ADHD	-	-	-	NA	-	-	FF		1200	Hom c.1007_1010del	p.Glu336Valfs*4	+	mega cisterna magna	NA
28	Oliveira J, *et al* 2015	Portuguese	F	+	NA	+	+	DCM	-	NA	NA	NA	NA	NA		299-1857	Hom c.1031+3G>C	Splice site affected		NA	NA
29	De Fuenmayor-Fernández De La Hoz CP, *et*	Bulgarian	F	-	-	mild	NA	-	-	NA	NA	NA	NA	NA		600-1500	Het c.701C>T	p.Ser234Leu	+	normal	NA

	al 2016																				
30	De Goede C, *et al* 2016	Indian	M	+	NA	+	NA	-	NA	NA	-	+	NA	-	apparent development delay after intercurrent gastrointestinal illness	1517	Hom c.722A>G	p.Asn241Ser	+	normal	involvement of the QF, SAR, and AM, spared of the GRA, HAM, AL, and AB.
31	Yis U, *et al* 2016	German		+	+	+	NA	NA	ichthyosis	-	NA	+	+	NA		406	Hom c.1031G>A	p.Arg344Gln		normal	+
32			M	+	+	+	NA	NA	nummular eczema	-	NA	-	+	NA		520	Hom c.1031G>A	p.Arg344Gln		normal	+
33	Brady L, *et al* 2016	canadian	F	-	-	-	-	-	NA	NA	NA	NA	NA	NA	rhabdomyolysis; episodes of weakness after stress or illness	400-1000	c.263C>T + c.950T>A	p.Pro88Leu + p.Leu317Gln	+	NA	NA
34			M	-	-	-	-	-	NA	NA	NA	NA	NA	NA	exercise intolerance	900-9000	c.263C>T + c.950T>A	p.Pro88Leu + p.Leu317Gln	+	NA	NA
35	Vanlander A V, *et al* 2016	Moroccan	M	+	+	+	NA	DCM	NA	NA	swallowing difficulties		NA	NA		5773	c.248dup	p.Arg84Profs*126	+		
	Marchet S, *et al* 2019	Italian	F	+	+	+	+	-	ichthyosis	anomalies in EEG	+	NA	+	NA		268	Hom c.565_568del	p.Phe189Glyfs*7	+	normal	diffused fatty infiltration of upper limbs
36			F	+	+	+	NA	+	ichthyosis	-	NA	NA	+	NA		944	Hom c.565_568del	p.Phe189Glyfs*7	+	NA	NA
37			F	+	+	+	+	-	-	+	NA	NA	+	NA	congenital neurosensorial deafness	180-250	Het c.140_146del + c.1066_1067del	p.Arg47Profs*21 + p.Trp356Valfs*72	+	NA	hypotrophy and fatty infiltration of lower limbs
38	Kutluk G, *et al* 2020	Turkish	F	+	+	-	autism	DCM	+	+	NA	-	-	-		500-1000	Hom c.818+1G>A	Splice site affected	+	normal	NA
39		Turkish	M	+	+	+	autism	-	ichthyosis	-	NA	-	+	-		500-1000	Hom c.1031+1G>A	Splice site affected	+	normal	NA
40	Chan SH, *et al* 2020	Chinese	F	+	NA	+	+	-	-	NA	NA	+	+	NA		821	Hom c.598del	p.Gln200Argfs*11	+	normal	generalized muscle involvement, spared AL and EDL
41			F	+	NA	+	-	-	-	NA	NA	+	+	NA		568	Hom c.598del	p.Gln200Argfs*11	NA	normal	generalized muscle involvement, spared AL and EDL
42	Bardhan M, *et al* 2021	Indian	F	+	+	NA	+	-	ichthyosis	NA	-	+	+	+		1447	Hom c.1027dup	p.Ser343Lysfs*86	+	NA	NA
43			F	+	+	+	+	-	dry	NA	+	+	+	+	hemangiomsa over abdomen and scalp; sleep disturbance	697	Hom c.224+1G > T	Splice site affected	NA	thinning of CC, enlarged CSF spaces in frontal convexities with prominent LV	NA
44			M	+	+	+	+	NA	dry	NA	+	+	+	NA		NA	Hom c.224+1G > T	Splice site affected	NA	NA	NA
45			M	+	+	+	+	-	-	NA	+	NA	-	+		NA	Hom c.1123C>T	p.Gln375*	NA	NA	NA
46			F	+	+	+	+	-	-	-	+	NA	-	+	disturbed sleep-wake cycle	2646	Hom c.581G>A	p.Arg194Gln	+	normal	posterior compartment muscle atrophy, spared AL
47	Magri F, *et al* 2022	Sri Lankan	M	+	+	+	+	-	NA	NA	NA	NA	NA	NA		560	c.1060G >C+ c.448-56_29del	p.Gly354Arg + p.Ser150Leufs*8	+	focal thinning of CC isthmus, mild asymmetry of temporal horn of LV	NA
48	Surucu Kara I, *et al* 2023	Turkish	M	+	+	+	+	-	-	NA	feeding difficulty	NA	+	+	sleep disturbance	335	Hom c.225-2A>T	Splice site affected	NA	normal	NA
49	Zemorshidi F, *et al* 2023	Iranian	M	+	+	+	-	-	-	-	NA	-	-	-		690-4809	Hom c.260T>C	p.Leu87Pro	+	normal	NA
50			M	+	-	+	-	-	vitiligo	+	NA	-	-	-		890-6186	Hom c.260T>C	p.Leu87Pro	+	normal	NA
51			F	NA	-	+	+	-	-	-	NA	-	-	+		1274	Hom c.722A>G	p.Asn241Ser	+	normal	NA
52			M	NA	+	+	+	-	-	-	NA	-	+	+		310-340	Hom c.382G>T	p.Glu128*	+	normal	NA
53			M	NA	-	+	+	-	-	+	NA	-	-	-		1639-1900	Hom c.539C>G	p.Pro180Arg	+	normal	NA
54			F	NA	-	+	+	-	-	-	NA	+	-	-		761	Hom c.1130G>T	p.Arg377Leu	+	NA	NA
55			M	+	+	+	+	DCM	ichthyosis	-	NA	-	+	-		1100	Hom c.1031+1G>A	Splice site affected	NA	NA	NA
56			F	-	-	+	+	-	-	-	NA	-	-	-		810	Hom c.392T>C	p.Leu131Pro	NA	NA	NA
57			M	+	+	+	+	-	-	+	NA	+	-	-		1200	Hom c.844dup	p.Cys282Leufs*2	NA	normal	NA

58			M	+	+	+	+	-	-	-	NA	-	-	-	776	Hom c.678-1G>C	Splice site affected	NA	normal	NA
59			F	+	+	+	+	-	+	-	NA	-	+	-	317	Hom c.737 − 1G>C	Splice site affected	NA	thinning of CC	NA
60			F	+	+	+	+	-	-	-	NA	-	+	-	331-651	Hom c.737 − 1G>C	Splice site affected	NA	normal	NA
61			M	+	+	+	+	-	-	+	NA	-	-	-	1452	Hom c.554_562del	p.Pro185_Trp187del	NA	NA	NA
62	Wu T, *et al* 2023	Chinese	F	+	+	NA	NA	-	NA	NA	NA	NA	+	NA	480	Hom c.225-2A>T	Splice site affected		normal	NA
63		Chinese	M	-	-	+	+	-	NA	NA	NA	NA	NA	NA	1646	Hom c.701C>T	p.Ser234Leu		normal	NA

ADHD, attention deficit hyperactivity disorder; DCM, dilated cardiomyopathy; LVD, left ventricular dilatation; DLVSF, decreased left ventricular systolic function; PDA, patent ductus arteriosus; ASD, atrial septal defect; MVP, mitral valve prolapsus; FF, facial features; ARDS, Acute Respiratory Distress Syndrome; URTI, upper respiratory tract infection; CSF, cerebrospinal fluid; CC, corpus callosum; LV, lateral ventricles; QF, quadriceps femoris; SAR, sartorius; AM, adductor magnus; GRA, gracilis; HAM, hamstrings; AL, adductor longus; AB, adductor brevis; EDL, extensor digitorum longus.

Supplementary Table S3. Clinical and demographic features for each variant type of *CHKB* related diseases

	**Frameshift**	**Missense/Inframe**	**Splicing**	**Nonsense**	**Total**	***P* value**
Patients	15	21	17	10	63	
Sex, male/female	7/8	10/11	10/7	4/6	31/32	
Age onset, median (years)	0.6 (0.1-1.3) [a]	3.0 (1.0-9.0) [a]	3.0 (0.9-4.0)	2.0 (0.9-3.3)	1.5 (0.6-3.0)	**0.023**
Motor delay, *n* (%)	15 (100)	12 (57.1)	17 (100)	8 (80)	52 (82.5)	**<0.001**
Speech delay, *n* (%)	13 (86.5)	11 (52.3)	16 (94.1)	8 (80)	48 (76.1)	**0.017**
Intellectual disability, *n* (%)	13 (86.5)	18 (85.7)	15 (88.2)	10 (100)	53 (88.9)	0.780
Behavioural problems, *n* (%)	9 (60)	8 (38.1)	13 (76.5)	5 (50)	35 (55.6)	0.134
Cardiac abnormalities, *n* (%)	5 (33.3)	2 (9.5)	6 (35.3)	3 (30)	14 (22.2)	0.208
Skin abnormalities, *n* (%)	5 (33.3)	6 (28.6)	11(64.7)	3 (30)	25 (39.7)	0.120
Seizure, *n* (%)	5 (33.3)	4 (19.0)	2 (11.8)	2 (20)	13 (20.1)	0.538
Bowel and bladder involvement, *n* (%)	2 (13.3)	1 (4.7)	3 (17.6)	2 (20)	8 (12.7)	0.515
Joint contractures, *n* (%)	4 (26.7)	3 (14.2)	1 (11.8)	0 (0)	9 (14.3)	0.222
Hypotonia, *n* (%)	10 (66.7)	3 (14.2)	11(64.7)	7 (70)	31 (49.2)	**0.001**
Facial weakness/features, *n* (%)	3 (33.3)	3 (14.2)	5 (29.4)	4 (40)	17 (27.0)	0.390
Serum creatine kinase level (IU/L)	694.5 (265.5-1200)	1363 (773.3-2460)	674.0 (470.3-1075)	511.0 (347.5-1710)	893.5 (476.8-1500)	0.052
MRI brain abnormality	5 (33.3)	0 (0)	3 (17.6)	3 (30)	11 (17.4)	**0.016**

Quantitative data, including age at onset and creatine kinase levels, were assessed for normality using the Shapiro–Wilk test and were non-normally distributed. These variables are therefore reported as median (first and third quartiles), and non-parametric tests were applied. Comparisons among the four groups were conducted using the Kruskal–Wallis test. When significant, post-hoc pairwise comparisons were performed using the Mann–Whitney *U* test with *P*-values adjusted using Dunn's correction. Categorical variables are presented as frequencies and percentages. Group comparisons were performed using the Chi-square test (for behavioral problems, skin abnormalities, and hypotonia) or the Fisher–Freeman–Halton exact test (for all other categorical variables). The Fisher–Freeman–Halton exact test was used when more than 20% of the contingency table cells had an expected count < 5. Significant *P*-values ($P < 0.05$) are shown in bold. [a]Age onset showed a statistically significant difference between missense/inframe and frameshift groups, with adjusted P = 0.016.
